# Sex Differences in the Relationship between Sleep Behavior, Fish Consumption, and Depressive Symptoms in the General Population of South Korea

**DOI:** 10.3390/ijerph14070789

**Published:** 2017-07-14

**Authors:** Atin Supartini, Taro Oishi, Nobuyuki Yagi

**Affiliations:** 1 Department of Behavioral and Health Sciences, Graduate School of Human-Environment Studies, Kyushu University, 6-1 Kasuga kouen, Kasuga City, Fukuoka Prefecture 816-8580, Japan; zamzabila@hotmail.com; 2 Faculty of Socio-Environmental Studies, Fukuoka Institute of Technology, 3-30-1, Wajiro-higashi, Higashi-ku, Fukuoka 811-0295, Japan; 3 Graduate School of Agricultural and Life Sciences, The University of Tokyo, 1-1-1, Yayoi, Bunkyo-ku, Tokyo 113-8657, Japan; yagi@fs.a.u-tokyo.ac.jp

**Keywords:** sleep timing, sleep quality, sleep latency, sleep duration, fish consumption, depressive symptoms

## Abstract

Sleep, fish consumption, and depression have a close relationship; however, the role of sex differences in sleep, fish consumption, and depression research is not yet well-established. This study aimed to examine whether the impact of bedtime, sleep-onset latency, sleep duration, sleep quality, and fish consumption on depressive symptoms differed in women and men. An online survey was conducted in South Korea with a stratified random sample of 600 participants between the ages of 20 and 69, whose gender and age were proportional to estimates of Korea’s general population. The 20-item Center for Epidemiologic Studies Depression Scale was used to measure depressive symptoms with a cut-off score of 16. The Pittsburgh Sleep Quality Index (PSQI) was applied to evaluate sleep timing, sleep-onset latency, sleep duration, and sleep quality. Our results indicated that late bedtime and short sleep duration were independently associated with depressive symptoms in women. Sleep-onset latency and poor sleep quality were independently associated with increased prevalence of depressive symptoms in both men and women. Higher fish consumption was significantly associated with decreased prevalence of depressive symptoms in men only. Our findings suggested the importance of a different approach for men and women in terms of promoting healthy sleep habits. In addition, higher fish consumption may be beneficial in the primary prevention of depression in Korean men. Further research is needed to confirm the findings from this cross-sectional study.

## 1. Introduction

Depression is a mental illness that affects approximately 23.1% of Korean males and 27.4% of females [1]. Depression has been claimed as one of the risk factors for lower quality of life [2], physical diseases [3], and suicide [4]. Unlike Japan, the suicide rates have continued to rise in Korea and is now the fourth leading cause of death [5]. Considering depression has such a large impact on society (e.g., healthcare costs, quality of life, and life expectancy) and is highly prevalent across all ages, it is important to understand the behavioral factors that may be linked to it. 

Sleep, diet, and exercise are three factors that play a significant mediating role in the development, progression, and treatment of depression [6]. While a large number of studies supported the benefit of exercise on reducing depression, studies on sleep and diet were less explored. A prospective study of Japanese adolescents revealed that sleep disturbance was significantly associated with poor mental health [7]. Chang et al. [8] found associations between poor sleep quality and depression in the elderly. A community health survey in Korea revealed that short sleep duration was associated with the increased prevalence of depression [9]. 

A recent meta-analysis study indicated that high-fish consumption could reduce the risk of depression [10]. A study conducted among Japanese employees showed that fish consumption was associated with resilience to depression [11]. Tanskanen et al. [12] found that the likelihood of having depressive symptoms was significantly higher among infrequent fish consumers than among frequent consumers. A longitudinal study of young adults aged seven to 15 years old from Australia suggested that fish consumption was associated with depression in women, but not in men [13]. 

Although previous studies have associated sleep and fish consumption with depression, several important issues need to be highlighted. First, previous studies on sleep and depression have mainly been conducted in Western countries; only a few studies have been conducted on the Korean population [8,14]. Due to differences in lifestyle and culture, findings obtained from Western countries might not be applicable to the Korean population. Second, while sleep disturbance such as insomnia is often the focus of sleep and depression research, aspects of sleep such as sleep quality and circadian chronotype are increasingly recognized for their substantial contribution to well-being. For example, the Netherlands Study of Depression and Anxiety revealed that late chronotype (evening-type of sleep) was associated with depressive disorder [15]. Studies conducted among workers [16] and freshmen [17] in Japan showed similar associations between late bedtime and depressive symptoms. Third, sex differences exist in sleep quality, duration, latency, and architecture in the general population [18]; however, the impact of sex differences in relation to sleep, fish consumption, and depression in the Korean population is not well-understood. 

In the current study, we attempted to overcome some of these limitations. Using a large, population-based national sample of Korean adults, we aimed to examine the associations between depressive symptoms, fish consumption, and various sleep behaviors—such as bedtime, sleep onset latency, sleep duration, and sleep quality—in men and women. We hypothesized that depressive symptoms would be higher in respondents who had less fish consumption, late bedtime, late wake-up time, prolonged sleep latency, short sleep duration, and poor sleep quality. To our knowledge, our study was the first to specifically examine sex differences of fish consumption and various sleep-behaviors in relation to depressive symptoms among the general population in Korea.

## 2. Materials and Methods 

### 2.1. Study Population

This study was performed over the course of one month from 23–31 January 2017 using a questionnaire developed by the authors. The online survey was conducted in Korea using a stratified random sample of 600 participants between the ages of 20 and 69, whose gender and age were proportional to an estimation of Korea’s general population. 

The participant had to fill out the online informed consent, which explained that the answers would be analyzed only for our research purposes and that there was no way to link the data to their identity when they registered as potential respondents for the survey company. All participants were assigned a random number in the survey by the server, and their names remained anonymous. 

### 2.2. Assessment of Depressive Symptoms

To assess the depressive symptoms, we used the Korean version of the Center for Epidemiological Depression Scale (CES-D), which had been previously validated in the Korean population [19]. The CES-D consists of 20 items with a total CES-D score ranged between 0–60. A score of 16 or higher indicated that the person is at risk of clinical depression [20].

### 2.3. Assessment of Sleep Behavior

Bedtime, wake-up time, sleep onset latency, sleep duration, and sleep quality were assessed by the Korean version of the Pittsburgh Sleep Quality Index (PSQI-K) [21]. The PSQI is a 19-item instrument that assesses sleep disturbances in seven areas during the prior month: subjective sleep quality, sleep latency, sleep duration, habitual sleep efficiency, sleep disturbances, use of sleep medications, and daytime dysfunctioning [22]. The global PSQI scores range from 0–21 and scores of five or higher indicate sleep disturbances [22]. However, in this study, we employed an 8.5-point threshold where people with 8.5 or more points were categorized as the poor sleep quality group, while people below 8.5 points were in the good sleep quality group. In the Korean version of the PSQI, a cutoff score of 8.5 represents a sensitivity of 0.943 and a specificity of 0.844, which is higher than the score of five in the original paper [21].

For this study, bedtime, sleep duration, and sleep onset latency were categorized into tertiles with references of early bedtime (≤23:00) for bedtime, (<6 h) for sleep duration, and 10 min or less for sleep onset latency.

### 2.4. Assessment of Fish Consumption

Using a questionnaire on health and the environment developed by the authors, participants were asked to estimate their fish consumption intake by choosing out of six response options ranging from never and less than once a year to almost every day. We categorized the fish consumption intake into three categories: frequently (a few times a week to almost every day), occasionally (a few times a month), and rarely (a few times a year or less). Participants were also asked whether they consumed smoked salmon which were categorized into three categories: frequently (a few times a week to almost every day), occasionally (a few times a month), and rarely (a few times a year or less).

### 2.5. Assessment of Other Variables

The questionnaire on health and the environment developed by the authors, comprised of questions regarding sociodemographic characteristics, lifestyle habits, and health conditions. Sociodemographic characteristics included in the analysis were age (20–29, 30–39, 40–49, 50–59, 60–69 years old), sex, living condition (living alone, living with family or friends), perceived health (very healthy, healthy, unhealthy), average monthly family income (10 million Korean won (KRW) or less, 20 million KRW or less, 30 million KRW or less, 40 million KRW or less, 50 million KRW or less, 50 million KRW or more), educational status (high school or lower, undergraduate level, graduate level or higher), job position (public official, company employee, business owner, self-employed, temporary employee, part-timer, student, housewife, househusband, unemployed, others), and “yes” or “no” responses to questions regarding religion, smoking habit, drinking habit, and exercise habit.

### 2.6. Statistical Analysis

Chi-square test was used to examine the differences in the proportion of sex, depressive symptoms cut-offs (absent and present), and sociodemographic characteristics. 

Multiple logistic regression analyses stratified by sex were used to analyze whether sleep behavior and fish consumption predict depressive symptoms. Three different models were tested to examine the association between sleep behavior and depression. First, we adjusted for age (Model 1). Previous research suggested that dietary factors and exercise may play a role in depression [6]. Therefore, in Model 2, fish consumption and exercise were mutually adjusted. In Model 3, we additionally adjusted for sociodemographic factors (education level, occupation, household income, living condition, and religion) and health behavior (perceived health status, drinking habit, and smoking habit). Likewise, three different models were tested to examine the association between fish consumption and depression. First, we adjusted for age (Model 1). In Model 2, sleep quality, exercise and smoked salmon were mutually adjusted. In Model 3, we additionally adjusted for socio-demographic factors (education level, occupation, household income, living condition, and religion) and health behavior (perceived health status, drinking habit, and smoking habit). A recent study indicated that omega-3 fatty acids have been shown to be associated with depression [23]. Therefore, we adjusted smoked salmon consumption in Model 2 and Model 3. Statistical analysis software SAS Version 9.2 (SAS Institute, Inc., Cary, NC, USA) was used for data analysis.

## 3. Results

### 3.1. Sociodemographic Characteristics of the Study Population

The socio-demographic characteristics, health behavior, and sleep behavior of the study population stratified by sex are summarized in Table 1. The distributions of several socio-demographic characteristics and health behavior tested in this study differed by gender. The significant differences could be seen in the education level (*p* < 0.0001), drinking habit (*p* = 0.0001), exercise habit (*p* = 0.01), sleep latency (*p* = 0.04), and sleep duration (*p* = 0.03).

**Figure 1:**
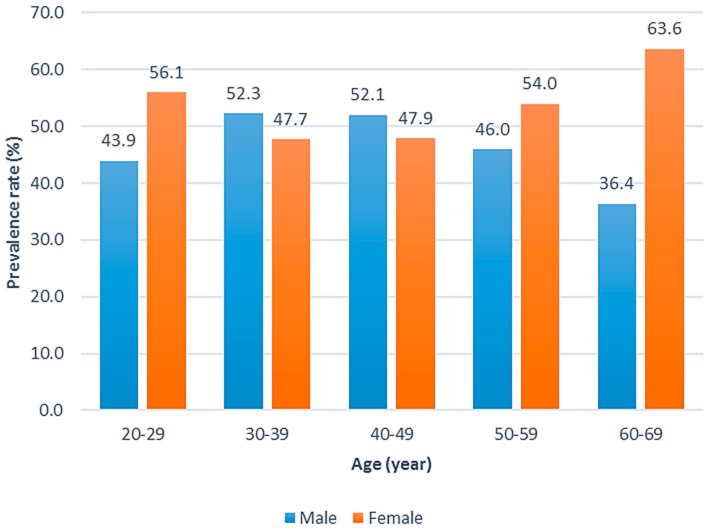
Prevalence of depressive symptoms in men and women.

The prevalence of depressive symptoms (defined by a cutoff score of 16) for the total population was 50% (men, 51%; women, 49%). In addition, as age increased, the prevalence of depressive symptoms tended to increase for women. In contrast, as age increased, the prevalence of depressive symptoms tended to decrease for men (Figure 1).

The relationship between depressive symptoms, socio-demographic characteristics, health behavior, and sleep behavior of the study population examined by cross tabulation are shown in Table 2. Depressive symptoms were significantly associated with living condition; whether the participant was living with family, friends or alone, income, fish consumption, and self-rated health. In addition, depressive symptoms tended to be higher among those who had poor sleep quality; bedtime, wake-up time, sleep latency, and sleep duration were significantly associated with depressive symptoms.

### 3.2. Association between Depressive Symptom and Sleep Behavior

Table 3 presents the results of a multiple logistic regression analysis where the regression of presence or absence of depressive symptoms on sleep behavior in men were analyzed. Sleep latency of more than 30 min and poor sleep quality were significantly associated with the increased prevalence of depressive symptoms. The association remained significant even when the model was adjusted for all other covariates, (OR: 1.93, 95% CI: 1.03–3.63) and (OR: 7.07, 95% CI: 3.03–16.50), respectively. Bedtime and sleep duration were not significantly associated with depressive symptoms.

Table 4 presents the results of a multiple logistic regression analysis where the regression of presence or absence of depressive symptoms on sleep behavior in women were analyzed. In females, a late bedtime of 24:30, sleep latency of more than 30 min, sleep duration of less than six hours, and poor sleep quality were significantly associated with increased prevalence of depressive symptoms. When we added other covariates, late bedtime, prolonged sleep latency, short sleep duration, and poor sleep quality remained significantly associated with depressive symptoms (OR: 2.36, 95% CI: 1.10–5.06), (OR: 2.97, 95% CI: 1.56–5.66), (OR: 4.63, 95% CI: 1.52–14.12), and (OR: 7.49, 95% CI: 2.69–20.88), respectively.

### 3.3. Association between Depressive Symptoms and Fish Consumption

Table 5 presents the results of a multiple logistic regression analysis where the regression of presence or absence of depressive symptoms on fish consumption in men were analyzed. Compared with those who consumed less fish, those who consumed fish frequently were significantly associated with having a lower odds ratio of depressive symptoms in the age adjusted model (OR: 0.41, 95% CI: 0.17–0.98). However, the association was not significant after adjusted for socio-demographic and health behavior variables (OR: 0.35, 95% CI: 0.11–1.10). Meanwhile, those who consumed fish occasionally were significantly associated with decreased odds of depressive symptoms even after adjustments for socio-demographic and health behavior variables (OR: 0.30, 95% CI: 0.09–0.97). Table 6 presents the results of a multiple logistic regression analysis where the regression of presence or absence of depressive symptoms on fish consumption in women were analyzed. In contrast to men, fish consumption in women did not show a significant association with depressive symptoms.

## 4. Discussion

The aim of our study was to detect gender-specific differences in the relationship between sleep behavior, fish consumption, and depressive symptoms. This study produced three main findings. First, sleep-onset latency and sleep quality were associated with increased odds of depressive symptoms, independent of all confounders, regardless of sex differences. Second, bedtime and sleep duration were significantly associated with an increased prevalence of depressive symptoms only in women. Third, higher fish consumption was significantly associated with decreased prevalence of depressive symptoms in men, but not in women.

Previous studies have reported a significant association between evening chronotype and depressive symptoms [15,24]. A study of almost 16,000 adolescents found an association between earlier parental set bedtime and depression [25]. Epidemiological studies conducted in Japan reported that late bedtime predicted depression in workers [16] and university students [17]. Consistent with those studies, our study found that those who had late bedtime habits were also at a higher risk of having depressive symptoms. However, our study found a significant association between late bedtime and depressive symptoms in women, but not in men. This could be due to the fact that men and women are different biologically and physiologically, and may underlie the differential risk for chronotype on depression [26]. These results highlight the importance for public health practitioners to promote early sleep timing for women in particular to prevent the development of depression. 

Sleep onset latency is one symptom of depression [27]. However, this association is thought to be bidirectional [28]. A previous epidemiology study among Japanese university students also identified that sleep-onset latency was associated with an increased prevalence of depressive symptoms [17]. These previous findings were consistent with our results which proved the importance of reducing sleep onset latency to lower the risk of depressive symptoms. Further research on factors related to sleep onset latency is warranted.

A recent meta-analysis on sleep duration and depression indicated that short and long sleep duration were significantly associated with increased risk of depression in adults [29]. Previous epidemiology studies among Japanese workers and university students also identified that short sleep duration was associated with increased prevalence of depressive symptoms [16,17]. Consistent with these findings, we found an increased prevalence of depressive symptoms among women with a short sleep duration (<6 h), but the association was not significant in men. The mechanism was unclear; however, a previous study found that poor sleep was strongly associated with high levels of psychological distress and greater feelings of hostility, depression, and anger [30]. Dysregulation of the serotonergic system is a potential mechanism that underlies the observed gender-specific relationship between sleep symptoms and depression [31].

The association between sleep quality and depression has been described in previous research [32,33]. Previous epidemiological studies revealed that sleep quality was associated with increased risk of depressive symptoms [17,32]. In a clinical study, sleep quality revealed itself as a significant determinant of onset of major depression [33]. In line with these findings, our study found that sleep quality predicted depressive symptoms in men and women, which demonstrated the importance of targeting sleep quality in men and women to prevent the development of depressive symptoms.

A longitudinal study of Australian young adults aged 7–15 years old suggested that fish consumption was associated with depression in women, but not in men [13]. In contrast, our study found that higher fish consumption was significantly associated with a decreased odds ratio of depressive symptoms in men. Similar to our finding, several cross-sectional studies and a prospective study have also observed a significant association between fish consumption and depression in men. For example, two cross-sectional studies conducted in Finland showed that higher fish consumption was associated with lower depression in men, but not in women [34]. In a prospective study using a large national US sample, men who consumed more fish were less likely to have depressive symptoms after a 10-year follow-up [35]. The reasons for these sex differences are not clear; however, age and hormonal differences may reflect the results in men and women. This result may be useful as a reference to reduce the risk of depression. 

The strengths of this study were the use of consecutive sampling with proportional sample size. However, the limitations of this study need to be addressed. First, we could not rule out recall bias in self-reported sleep behavior and depressive symptoms. Second, its cross-sectional design did not allow us to infer causality or specify the direction of the effect. Third, although we used reliable and valid measures of depressive symptoms, these measures did not constitute a clinical diagnosis of depressive disorders. Fourth, an internet survey can be subject to significant biases resulting from under-coverage and nonresponse. However, we have reduced the bias by using random sample strategy and we only included the respondents who completed the survey.

## 5. Conclusions

The present study found that late bedtime and short sleep duration were independently associated with depressive symptoms in women. Sleep-onset latency and poor sleep quality were independently associated with increased prevalence of depressive symptoms in both men and women. Higher fish consumption was significantly associated with decreased prevalence of depressive symptoms in men. Our findings suggest the importance of developing specific sleep interventions that are particularly effective to target men and women to prevent the development of depressive symptoms. In addition, higher fish consumption may be beneficial in the primary prevention of depression among men in Korean population. Further prospective study is needed to confirm the findings from this cross-sectional study. 

## Figures and Tables

**Table 1:** The sociodemographic characteristics, health behavior, and sleep behavior of the study population stratified by sex.

Variables	Component	Total % (*n* = 600)	Male % (*n* = 306)	Female % (*n* = 294)	*p*-Value
Age	20–29	18.8	19.6	18.0	0.982
	30–39	21.3	21.6	21.1	
	40–49	23.7	23.5	23.8	
	50–59	22.5	22.2	22.8	
	60–69	13.7	13.1	14.3	
Occupation	Public official	2.8	4.3	1.4	<0.0001
	Company employee	48.2	54.3	41.8	
	Business owner/self-employed	8.8	13.4	4.1	
	Temporary employee	4.0	4.6	3.4	
	Casual worker	3.5	2.0	5.1	
	Student	6.8	9.5	4.1	
	Housewife	15.7	0	32.0	
	Unemployed	5.3	5.6	5.1	
	Other	4.8	6.5	3.1	
Religion	Yes	42.6	41.5	43.7	0.594
	No	57.4	58.5	56.3	
Education level	High school and lower	20.6	15.5	25.9	<0.0001
	Undergraduate level	68.8	69.6	67.9	
	Graduate level	10.6	14.9	6.1	
Living condition	With family or friends	89.0	11.4	10.5	0.727
	Alone	11.0	88.6	89.5	
Income	10,000,000 KRW or less	4.2	3.8	4.6	0.874
	20,000,000 KRW or less	5.4	5.1	5.6	
	30,000,000 KRW or less	11.9	13.3	10.5	
	40,000,000 KRW or less	13.0	13.7	12.3	
	50,000,000 KRW or less	16.1	16.4	15.8	
	More than 50,000,000 KRW	49.5	47.8	51.2	
Perceived health	Very healthy	12.3	14.7	9.9	0.172
	Healthy	67.0	66.0	68.0	
	Unhealthy	20.7	19.3	22.1	
Drinking habit	Yes	71.7	80.4	62.6	<0.0001
	No	28.3	19.6	37.4	
Smoking habit	Yes	27.3	42.5	11.6	<0.0001
	No	72.7	57.5	88.4	
Exercise habit	Yes	27.7	32.0	23.1	0.01
	No	72.23	68.0	76.9	
Fish consumption	Frequently	48.7	50.0	47.3	0.798
	Occasionally	41.5	40.5	42.5	
	Rarely	9.8	9.5	10.2	
Smoked salmon consumption	Frequently	72.2	74.2	70.1	0.437
	Occasionally	22.3	21.2	23.4	
	Rarely	5.5	4.6	6.5	
Bedtime	23:00 and earlier	33.5	36.6	30.3	0.257
	23:01 to 24:30	39.3	37.3	41.5	
	Later than 24:30	27.2	26.1	28.2	
Sleep latency	10 min and less	41.7	45.75	37.4	0.04
	11 min to less than 30 min	23.3	23.86	22.8	
	More than 30 min	35	30.39	39.8	
Sleep duration	Less than 6 h	13.7	16.3	10.9	0.03
	6 to 8 h	81.0	80.1	82.0	
	More than 8 h	5.3	3.6	7.1	
Sleep quality	Good	83.7	83.7	83.7	0.997
	Poor	16.3	16.3	16.3	
Depressive symptoms	Present	50.0	46.4	53.7	0.07
	Absent	50.0	53.6	46.3	

**Table 2:** The relationship between depressive symptoms, sociodemographic characteristics, and health behavior of the study population.

Variables	Component	Total % (*n* = 600)	Depressive Symptoms		*p*-Value
			Absent	Present	
Sex	Male	51.0	54.7	47.3	0.07
	Female	49.0	45.3	52.7	
Age	20–29	18.8	15.7	22.0	0.133
	30–39	21.3	21.0	21.7	
	40–49	23.7	23.0	24.3	
	50–59	22.5	24.0	21.0	
	60–69	13.7	16.3	11.0	
Occupation	Public official	2.8	3.3	2.3	0.07
	Company employee	48.2	47.0	49.3	
	Business owner/self-employed	8.8	9.7	8.0	
	Temporary employee	4.0	4.3	3.7	
	Casual worker	3.5	3.3	3.7	
	Student	6.8	6.0	7.7	
	Housewife	15.7	19.0	12.3	
	Unemployed	5.3	2.7	8.0	
	Other	4.8	4.7	5.0	
Religion	Yes	42.6	45.1	40.1	0.229
	No	57.4	55.0	59.9	
Education level	High school and lower	20.6	18.7	22.6	0.16
	Undergraduate level	68.8	68.6	69.0	
	Graduate level	10.6	12.7	8.4	
Living condition	With family or friends	11.0	93.0	85.0	0.002
	Alone	89.0	7.0	15.0	
Income	10,000,000 KRW or less	4.2	2.1	6.3	0.018
	20,000,000 KRW or less	5.4	4.1	6.6	
	30,000,000 KRW or less	11.9	9.6	14.3	
	40,000,000 KRW or less	13.0	12.7	13.2	
	50,000,000 KRW or less	16.1	17.2	15.0	
	More than 50,000,000 KRW	49.5	54.3	44.6	
Perceived health	Very healthy	12.3	15.3	9.3	<0.0001
	Healthy	67.0	73.7	60.3	
	Unhealthy	20.7	11.0	30.3	
Drinking habit	Yes	71.7	71.7	71.7	1
	No	28.3	28.3	28.3	
Smoking habit	Yes	27.3	25.7	29.0	0.36
	No	72.7	74.3	71.0	
Exercise habit	Yes	27.7	28.3	27.0	0.715
	No	72.3	71.7	73.0	
Fish consumption	Frequently	48.7	51.7	45.7	0.049
	Occasionally	41.5	41.3	41.7	
	Rarely	9.8	7.0	12.7	
Smoked salmon consumption	Frequently	72.2	73.3	71.0	0.568
	Occasionally	22.3	20.7	24.0	
	Rarely	5.5	6.0	5.0	
Bedtime	23:00 and earlier	33.5	37.0	30.0	0.047
	23:01 to 24:30	39.3	40.0	38.7	
	Later than 24:30	27.2	23.0	31.3	
Sleep latency	10 min and less	41.7	49.3	34.0	<0.0001
	11 min to less than 30 min	23.3	25.3	21.3	
	More than 30 min	35.0	25.3	44.7	
Sleep duration	Less than 6 h	13.7	10.0	17.3	0.02
	6 to 8 h	81.0	85.3	76.7	
	More than 8 h	5.3	4.7	6.0	
Sleep quality	Good	83.7	94.7	72.7	<0.0001
	Poor	16.3	5.3	27.3	

**Table 3:** Multiple logistic regression of sleep behavior related to depressive symptoms in men.

Variables		Model 1		Model 2		Model 3	
		Odds Ratio (CI)		Odds Ratio (CI)		Odds Ratio (CI)	
Bedtime							
	23:00 and earlier	1		1		1	
	23:01 to 24:30	0.84	(0.50–1.43)	0.81	(0.47–1.37)	0.77	(0.42–1.42)
	Later than 24:30	0.81	(0.45–1.48)	0.79	(0.43–1.44)	0.76	(0.38–1.52)
Sleep latency							
	10 min and less	1		1		1	
	11 min to less than 30 min	1.24	(0.70–2.21)	1.24	(0.69–2.23)	1.12	(0.58–2.17)
	More than 30 min	2.26	(1.32–3.87)	2.19	(1.27–3.78)	1.93	(1.03–3.63)
Sleep duration							
	Less than 6 h	1.43	(0.77–2.65)	1.48	(0.80–2.75)	1.61	(0.79–3.27)
	6 to 8 h	1		1		1	
	More than 8 h	2.82	(0.72–11.13)	2.60	(0.64–10.48)	2.52	(0.41–15.31)
Sleep quality							
	Good	1		1		1	
	Poor	6.24	(2.97–13.01)	6.25	(2.96–13.18)	7.07	(3.03–16.50)

Model 1: adjusted for age; Model 2: adjusted for age, fish consumption, and exercise; Model 3: Model 2 + adjusted for socio-demographic and health behavior variables.

**Table 4:** Multiple logistic regression of sleep behavior related to depressive symptoms in women.

Variables		Model 1		Model 2		Model 3	
		Odds Ratio (CI)		Odds Ratio (CI)		Odds Ratio (CI)	
Bedtime							
	23:00 and earlier	1		1		1	
	23:01 to 24:30	1.63	(0.93–2.84)	1.63	(0.93–2.84)	1.58	(0.83–3.04)
	Later than 24:30	3.10	(1.65–5.84)	3.09	(1.64–5.85)	2.36	(1.10–5.06)
Sleep latency							
	10 min and less	1		1		1	
	11 min to less than 30 min	1.05	(0.57–1.95)	1.06	(0.57–1.97)	1.01	(0.48–2.14)
	More than 30 min	2.57	(1.49–4.43)	2.67	(1.53–4.63)	2.97	(1.56–5.66)
Sleep duration							
	Less than 6 h	5.50	(2.12–14.26)	5.45	(2.09–14.23)	4.63	(1.52–14.12)
	6 to 8 h	1		1		1	
	More than 8 h	0.72	(0.28–1.80)	0.71	(0.28–1.80)	0.71	(0.24–2.09)
Sleep quality							
	Good	1		1		1	
	Poor	8.28	(3.37–20.34)	8.40	(3.39–20.81)	7.49	(2.69–20.88)

Model 1: adjusted for age; Model 2: adjusted for age, fish consumption, and exercise; Model 3: Model 2 + adjusted for socio-demographic and health behavior variables.

**Table 5:** Multiple logistic regression of fish consumption related to depressive symptoms in men.

Variables		Model 1		Model 2		Model 3	
		Odds Ratio (CI)		Odds Ratio (CI)		Odds Ratio (CI)	
Fish consumption							
	Frequently	0.41	(0.17–0.98)	0.40	(0.16–0.99)	0.35	(0.11–1.10)
	Occasionally	0.37	(0.16–0.87)	0.35	(0.14–0.88)	0.30	(0.09–0.97)
	Rarely	1		1		1	

Model 1: adjusted for age; Model 2: adjusted for age, sleep quality, exercise, and smoked salmon; Model 3: Model 2 + adjusted for socio-demographic and health behavior variables.

**Table 6:** Multiple logistic regression of fish consumption related to depressive symptoms in women.

Variables		Model 1		Model 2		Model 3	
		Odds Ratio (CI)		Odds Ratio (CI)		Odds Ratio (CI)	
Fish consumption							
	Frequently	0.81	(0.36–1.84)	1.05	(0.43–2.53)	1.59	(0.52–4.90)
	Occasionally	0.69	(0.31–1.54)	1.02	(0.41–2.53)	1.52	(0.46–4.97)
	Rarely	1		1		1	

Model 1: adjusted for age; Model 2: adjusted for age, sleep quality, exercise, and smoked salmon; Model 3: Model 2 + adjusted for socio-demographic and health behavior variables.

## References

[1] Cho M.J., Nam J.J., Suh G.H. (1998). Prevalence of symptoms of depression in a nationwide sample of Korean adults. Psychiatry Res..

[2] Hasche L.K., Morrow-Howell N., Proctor E.K. (2010). Quality of Life Outcomes for Depressed and Non-Depressed Older Adults in Community Long Term Care. Am. J. Geriatr. Psychiatry  Off. J. Am. Assoc. Geriatr. Psychiatry.

[3] Goodwin G.M. (2006). Depression and associated physical diseases and symptoms. Dialogues Clin. Neurosci..

[4] Nanayakkara S., Misch D., Chang L., Henry D. (2013). Depression and exposure to suicide predict suicide attempt. Depress Anxiety.

[5] Kaneita Y., Ohida T., Osaki Y., Tanihata T., Minowa M., Suzuki K., Wada K., Kanda H., Hayashi K. (2006). Insomnia among Japanese adolescents: A nationwide representative survey. Sleep.

[6] Lopresti A.L., Hood S.D., Drummond P.D. (2013). A review of lifestyle factors that contribute to important pathways associated with major depression: Diet, sleep and exercise. J. Affect. Disord..

[7] Kaneita Y., Yokoyama E., Harano S., Tamaki T., Suzuki H., Munezawa T., Nakajima H., Asai T., Ohida T. (2009). Associations between sleep disturbance and mental health status: A longitudinal study of Japanese junior high school students. Sleep Med..

[8] Chang K.J., Son S.J., Lee Y., Back J.H., Lee K.S., Lee S.J., Chung Y.K., Lim K.Y., Noh J.S., Kim H.C. (2014). Perceived sleep quality is associated with depression in a Korean elderly population. Arch. Gerontol. Geriatr..

[9] Ryu S.Y., Kim K.S., Han M.A. (2011). Factors Associated with Sleep Duration in Korean Adults: Results of a 2008 Community Health Survey in Gwangju Metropolitan City, Korea. J. Korean Med. Sci..

[10] Li F., Liu X., Zhang D. (2016). Fish consumption and risk of depression: A meta-analysis. J. Epidemiol. Community Health.

[11] Yoshikawa E., Nishi D., Matsuoka Y. (2015). Fish consumption and resilience to depression in Japanese company workers: A cross-sectional study. Lipids Health Dis..

[12] Tanskanen A., Hibbeln J.R., Tuomilehto J., Uutela A., Haukkala A., Viinamäki H., Lehtonen J., Vartiainen E. (2001). Fish consumption and depressive symptoms in the general population in Finland. Psychiatr. Serv..

[13] Smith K.J., Sanderson K., McNaughton S.A., Gall S.L., Dwyer T., Venn A.J. (2014). Longitudinal associations between fish consumption and depression in young adults. Am. J. Epidemiol..

[14] Park J.H., Yoo M.S., Bae S.H. (2013). Prevalence and predictors of poor sleep quality in Korean older adults. Int. J. Nurs. Pract..

[15] Antypa N., Vogelzangs N., Meesters Y., Schoevers R., Penninx B.W. (2016). Chronotype associations with depression and anxiety disorders in a large cohort study. Depress Anxiety.

[16] Sakamoto N., Nanri A., Kochi T., Tsuruoka H., Pham N.M., Kabe I., Matsuda S., Mizoue T. (2013). Bedtime and sleep duration in relation to depressive symptoms among Japanese workers. J. Occup. Health.

[17] Supartini A., Honda T., Basri N.A., Haeuchi Y., Chen S., Ichimiya A., Kumagai S. (2016). The Impact of sleep timing, sleep duration, and sleep quality on depressive symptoms and suicidal ideation amongst Japanese freshmen: The EQUSITE Study. Sleep Disord..

[18] Ohayon M.M., Reynolds C.F., Dauvilliers Y. (2013). Excessive sleep duration and quality of life. Ann. Neurol..

[19] Cho M.J., Kim K.H. (1993). Diagnostic validity of the CES-D (Korean version) in the assessment of DSM-III-R major depression. J. Korean Neuropsychiatr. Assoc..

[20] Radloff L.S. (1977). The CES-D Scale: A self-report depression scale for research in the general population. Appl. Psychol. Meas..

[21] Sohn S., Kim D., Lee M., Cho Y.W. (2012). The reliability and validity of the Korean version of the Pittsburg Sleep Quality Index. Sleep Breath..

[22] Buysse D.J., Reynolds C.F., Monk T.H., Berman S.R., Kupfer D.J. (1989). The Pittsburgh sleep quality index: A new instrument for psychiatric practice and research. Psychiatry Res..

[23] Logan A.C. (2004). Omega-3 fatty acids and major depression: A primer for mental health professional. Lipids Health Dis..

[24] Hirata F.C., Lima M.C.O., de Bruin V.M.S., Nóbrega P.R., Wenceslau G.P., de Bruin P.F. (2007). Depression in medical school: The influence of morningness–eveningness. Chronobiol. Int..

[25] Gangwisch J.E., Babiss L.A., Malaspina D., Turner J.B., Zammit G.K., Posner K. (2010). Earlier parental set bedtimes as a protective factor against depression and suicidal ideation. Sleep.

[26] Mallampalli M.P., Carter C.L. (2014). Exploring Sex and Gender Differences in Sleep Health: A Society for Women’s Health Research Report. J. Women’s Health.

[27] Nutt D., Wilson S., Paterson L. (2008). Sleep disorders as core symptoms of depression. Dialogues Clin. Neurosci..

[28] Franzen P.L., Buysse D.J. (2008). Sleep disturbances and depression: Risk relationships for subsequent depression and therapeutic implications. Dialogues Clin. Neurosci..

[29] Zhai L., Zhang H., Zhang D. (2015). Sleep duration and depression among adults: A meta-analysis of prospective studies. Depress Anxiety.

[30] Suarez E.C. (2008). Self-reported symptoms of sleep disturbance and inflammation, coagulation, insulin resistance and psychosocial distress: Evidence for gender disparity. Brain Behav. Immun..

[31] Voderholzer U., Hornyak M., Thiel B., Huwig-Poppe C., Kiemen A., König A., Backhaus J., Riemann D., Berger M., Hohagen F. (1998). Impact of experimentally induced serotonin deficiency by tryptophan depletion on sleep EEC in healthy subjects. Neuropsychopharmacology.

[32] Selvi Y., Aydin A., Boysan M., Atli A., Agargun M.Y., Besiroglu L. (2010). Associations between chronotype, sleep quality, suicidality, and depressive symptoms in patients with major depression and healthy controls. Chronobiol. Int..

[33] Norra C., Kummer J., Boecker M., Skobel E., Schauerte P., Wirtz M., Gauggel S., Forkmann T. (2012). Poor sleep quality is associated with depressive symptoms in patients with heart disease. Int. J. Behav. Med..

[34] Suominen-Taipale A.L., Partonen T., Turunen A.W., Männistö S., Jula A., Verkasalo P.K. (2010). Fish consumption and omega-3 polyunsaturated fatty acids in relation to depressive episodes: A cross-sectional analysis. PLoS ONE.

[35] Li Y., Dai Q., Ekperi L.I., Dehal A., Zhang J. (2011). Fish consumption and severely depressed mood, findings from the first national nutrition follow-up study. Psychiatry Res..

